# 1,3-Di-4-pyrid­ylpropane–2-hydroxy­benzene-1,4-dicarboxylic acid (1/2)

**DOI:** 10.1107/S1600536808037835

**Published:** 2008-11-20

**Authors:** Jian-Hua Qin, Er-Jun Hao, Jian-Ge Wang

**Affiliations:** a College of Chemistry and Chemical Engineering, Luoyang Normal University, Luoyang 471022, People’s Republic of China; b College of Chemistry and Environmental Science, Henan Normal University, Xinxiang, 453007, People’s Republic of China

## Abstract

In the title compound, C_13_H_14_N_2_·2C_8_H_6_O_5_, which crystallized in the monoclinic *C*2/*c* space group, the 1,3-bis­(4-pyrid­yl)propane mol­ecules and 2-hydr­oxy-1,4-benzene­dicarboxylic acid mol­ecules are alternately linked by O—H⋯N and O—H⋯O hydrogen bonds into herringbone/zigzag chains.

## Related literature

For general background, see: Bowers *et al.* (2005 ▶); Mukherjee *et al.* (2004 ▶). For the substitution of bromine for hydroxyl, see: Chen & Tong (2007 ▶); Zhang (2005 ▶).
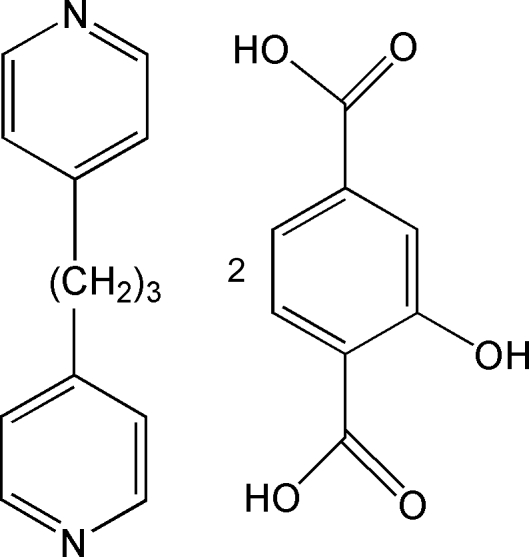


## Experimental

### 

#### Crystal data

C_13_H_14_N_2_·2C_8_H_6_O_5_
*M* *_r_* = 562.52
Monoclinic, 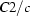

*a* = 22.939 (11) Å
*b* = 4.781 (2) Å
*c* = 24.163 (11) Å
β = 96.542 (6)°
*V* = 2633 (2) Å^3^
*Z* = 4
Mo *K*α radiation
μ = 0.11 mm^−1^
*T* = 291 (2) K
0.35 × 0.19 × 0.05 mm

#### Data collection

Bruker CCD area-detector diffractometer
Absorption correction: multi-scan (*SADABS*; Bruker, 1997 ▶) *T*_min_ = 0.963, *T*_max_ = 0.994
9178 measured reflections
2444 independent reflections
1335 reflections with *I* > 2σ(*I*)
*R*_int_ = 0.049

#### Refinement

*R*[*F* ^2^ > 2σ(*F* ^2^)] = 0.056
*wR*(*F* ^2^) = 0.173
*S* = 1.03
2444 reflections
187 parameters
H-atom parameters constrained
Δρ_max_ = 0.39 e Å^−3^
Δρ_min_ = −0.18 e Å^−3^

### 

Data collection: *SMART* (Bruker, 1997 ▶); cell refinement: *SAINT* (Bruker, 1997 ▶); data reduction: *SAINT*; program(s) used to solve structure: *SHELXS97* (Sheldrick, 2008 ▶); program(s) used to refine structure: *SHELXL97* (Sheldrick, 2008 ▶); molecular graphics: *SHELXTL* (Sheldrick, 2008 ▶); software used to prepare material for publication: *SHELXTL*.

## Supplementary Material

Crystal structure: contains datablocks global, I. DOI: 10.1107/S1600536808037835/ww2129sup1.cif

Structure factors: contains datablocks I. DOI: 10.1107/S1600536808037835/ww2129Isup2.hkl

Additional supplementary materials:  crystallographic information; 3D view; checkCIF report

## Figures and Tables

**Table 1:** Hydrogen-bond geometry (Å, °)

*D*—H⋯*A*	*D*—H	H⋯*A*	*D*⋯*A*	*D*—H⋯*A*
O4—H4⋯O5^i^	0.82	1.82	2.631 (3)	172
O2—H2⋯N1	0.82	1.75	2.568 (3)	174
O1—H1⋯O3	0.82	1.79	2.516 (3)	147

Symmetry code: (i) 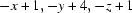.

**Fig. 1.**
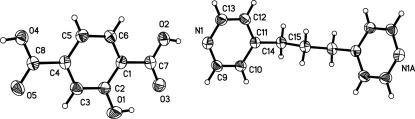
A view of the title compound, showing 30% probability displacement ellipsoids. Symmetry code: (A) -x, y, 1/2 - z.

**Fig. 2.**
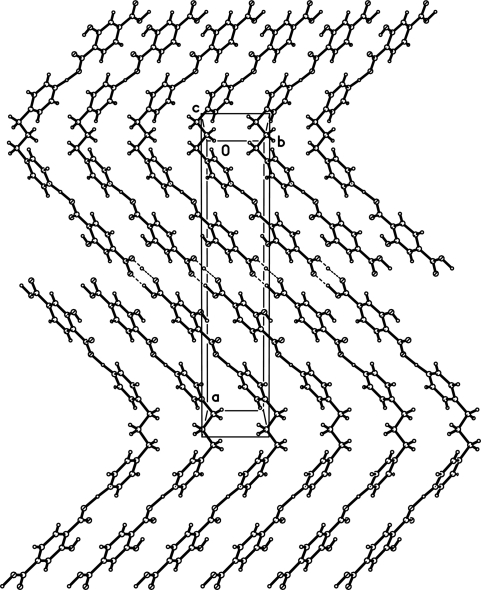
The unit-cell packing of the title compound, showing the hydrogen bonding interactions.

## supplementary crystallographic information

### Comment

The aromatic dicarboxylate are used extensively in the synthesis of coordination polymers (Mukherjee *et al.*, 2004) and the generation of hydrogen-bonding arrays of organic co-crystals (Bowers *et al.*, 2005). The 2-bromo-1,4-benzenedicarboxylic acid, possesses several interesting characteristics: (*a*) it has two carboxyl groups which may be completely or partially deprotonated, inducing rich coordination modes and allowing interesting structures with higher dimensions; (*b*) it can act not only as hydrogen-bond acceptor but also as hydrogen-bond donor, depending upon the number of deprotonated carboxyl groups. To propagate non-covalent interactions only along one direction, each unit must have two-point interactions with two adjacent neighbors. Dicarboxylic acids epitomize this model and exhibits a two-point contact per unit that can result in 1-D hydrogen bonding networks.

The crystal structure of the title compound comprises half of a 1,3-bis(4-pyridyl)propane (bpp) molecule and a 2-hydroxy-1,4-benzenedicarboxylic acid molecule per asymmetric unit (Fig. 1). One bpp molecule connects two adjacent 2-hydroxy-1,4-benzenedicarboxylic acid molecules *via* O—H···N hydrogen bonds, and complementary *via* O—H···O hydrogen bonds between adjacent dicarboxylic acid molecules, which extend into an one-dimensional herringbone/zigzag chain structure (Fig. 2). The dihedral angle between two pyridyl rings is 85.551 (11)°, and thus incorporation into supramolecular architecture imparts significant conformation change in the bpp molecule. The 1-D chain structure seems to suggest that the CH_2_ chain in pyridyl bases is not an inert spacer but could have a conformational and structural role in controlling the supramolecular architecture.

### Experimental

1,3-Bis(4-pyridyl)propane (bpp) (0.5 mmol), 2-bromo-1,4-benzenedicarboxylic acid (1.0 mmol), and KOH (0.5 mmol) were added to water (12 ml) in a Teflon-lined stainless steel reactor. The mixture was heated at 435 K for 3 d, and then slowly cooled down to room temperature. Colorless crystals of the title compound were obtained. Elemental analysis – found: C, 61.81%; H, 4.56%; N, 4.92%; calc. for C_29_H_26_N_2_ O_10_: C, 61.86%; H, 4.62%; N, 4.98%. Note the substitution of bromine for hydroxyl in the formation of title compound, which commonly happened under hydro(solvo)thermal conditions (Chen & Tong, 2007; Zhang, 2005).

### Refinement

H atoms were positioned geometrically and treated as riding, with C—H bonding lengths constrained to 0.93 (aromatic CH), or 0.97Å (methylene CH_2_), and O—H=0.82Å, and with *U*_iso_(H) = 1.2*U*_eq_(C) or *U*_iso_(H) = 1.5*U*_eq_(O).

### Crystal data

**Table:**

C_13_H_14_N_2_·2C_8_H_6_O_5_	*F*_000_ = 1176
*M**_r_* = 562.52	*D*_x_ = 1.419 Mg m^−^^3^
Monoclinic, *C*2/*c*	Mo *K*α radiation λ = 0.71073 Å
Hall symbol: -C 2yc	Cell parameters from 1102 reflections
*a* = 22.939 (11) Å	θ = 3.4–23.3º
*b* = 4.781 (2) Å	µ = 0.11 mm^−^^1^
*c* = 24.163 (11) Å	*T* = 291 (2) K
β = 96.542 (6)º	Block, colorless
*V* = 2633 (2) Å^3^	0.35 × 0.19 × 0.05 mm
*Z* = 4	

### Data collection

**Table:**

Bruker SMART CCD area-detector diffractometer	2444 independent reflections
Radiation source: fine-focus sealed tube	1335 reflections with *I* > 2σ(*I*)
Monochromator: graphite	*R*_int_ = 0.049
*T* = 291(2) K	θ_max_ = 25.5º
ω & φ scans	θ_min_ = 2.6º
Absorption correction: multi-scan(SADABS; Bruker, 1997)	*h* = −27→27
*T*_min_ = 0.963, *T*_max_ = 0.994	*k* = −5→5
9178 measured reflections	*l* = −29→28

### Refinement

**Table:**

Refinement on *F*^2^	Secondary atom site location: difference Fourier map
Least-squares matrix: full	Hydrogen site location: inferred from neighbouring sites
*R*[*F*^2^ > 2σ(*F*^2^)] = 0.056	H-atom parameters constrained
*wR*(*F*^2^) = 0.173	*w* = 1/[σ^2^(*F*_o_^2^) + (0.0772*P*)^2^ + 1.353*P*] where *P* = (*F*_o_^2^ + 2*F*_c_^2^)/3
*S* = 1.03	(Δ/σ)_max_ < 0.001
2444 reflections	Δρ_max_ = 0.39 e Å^−^^3^
187 parameters	Δρ_min_ = −0.18 e Å^−^^3^
Primary atom site location: structure-invariant direct methods	Extinction correction: none

### Special details

**Table:**

Geometry. All e.s.d.'s (except the e.s.d. in the dihedral angle between two l.s. planes) are estimated using the full covariance matrix. The cell e.s.d.'s are taken into account individually in the estimation of e.s.d.'s in distances, angles and torsion angles; correlations between e.s.d.'s in cell parameters are only used when they are defined by crystal symmetry. An approximate (isotropic) treatment of cell e.s.d.'s is used for estimating e.s.d.'s involving l.s. planes.
Refinement. Refinement of *F*^2^ against ALL reflections. The weighted *R*-factor *wR* and goodness of fit *S* are based on *F*^2^, conventional *R*-factors *R* are based on *F*, with *F* set to zero for negative *F*^2^. The threshold expression of *F*^2^ > σ(*F*^2^) is used only for calculating *R*-factors(gt) *etc*. and is not relevant to the choice of reflections for refinement. *R*-factors based on *F*^2^ are statistically about twice as large as those based on *F*, and *R*- factors based on ALL data will be even larger.

### Fractional atomic coordinates and isotropic or equivalent isotropic displacement parameters (Å^2^)

**Table:**

	*x*	*y*	*z*	*U*_iso_*/*U*_eq_	Occ. (<1)
O1	0.38415 (11)	1.0471 (6)	0.32590 (9)	0.0857 (9)	
H1	0.3557	0.9516	0.3141	0.129*	
O2	0.24082 (9)	0.8169 (5)	0.40221 (9)	0.0642 (7)	
H2	0.2203	0.6976	0.3852	0.096*	
O3	0.29118 (9)	0.7716 (5)	0.32941 (9)	0.0695 (7)	
O4	0.43663 (9)	1.8252 (5)	0.51792 (9)	0.0666 (7)	
H4	0.4624	1.9400	0.5274	0.100*	
O5	0.48854 (9)	1.7794 (5)	0.44642 (10)	0.0679 (7)	
N1	0.17662 (11)	0.4242 (5)	0.35544 (11)	0.0516 (7)	
C1	0.32537 (12)	1.0947 (6)	0.40090 (12)	0.0467 (7)	
C2	0.37382 (13)	1.1721 (6)	0.37430 (13)	0.0539 (8)	
C3	0.41262 (13)	1.3722 (6)	0.39812 (13)	0.0568 (8)	
H3	0.4451	1.4215	0.3805	0.068*	
C4	0.40338 (12)	1.4989 (6)	0.44770 (12)	0.0470 (7)	
C5	0.35515 (13)	1.4263 (6)	0.47459 (13)	0.0535 (8)	
H5	0.3486	1.5125	0.5078	0.064*	
C6	0.31687 (13)	1.2222 (6)	0.45088 (13)	0.0551 (8)	
H6	0.2849	1.1702	0.4690	0.066*	
C7	0.28337 (13)	0.8782 (6)	0.37506 (14)	0.0527 (8)	
C8	0.44604 (13)	1.7142 (6)	0.47143 (13)	0.0493 (8)	
C9	0.18299 (12)	0.3070 (7)	0.30666 (13)	0.0534 (8)	
H9	0.2147	0.3584	0.2881	0.064*	
C10	0.14366 (12)	0.1103 (6)	0.28276 (12)	0.0516 (8)	
H10	0.1493	0.0298	0.2488	0.062*	
C11	0.09577 (12)	0.0334 (6)	0.30954 (12)	0.0449 (7)	
C12	0.09152 (13)	0.1551 (7)	0.36110 (13)	0.0570 (8)	
H12	0.0609	0.1056	0.3813	0.068*	
C13	0.13223 (14)	0.3479 (7)	0.38230 (13)	0.0575 (8)	
H13	0.1285	0.4277	0.4168	0.069*	
C14	0.04828 (11)	−0.1557 (6)	0.28235 (12)	0.0499 (8)	
H14A	0.0318	−0.2651	0.3106	0.060*	
H14B	0.0648	−0.2837	0.2572	0.060*	
C15	0.0000	0.0194 (8)	0.2500	0.0498 (11)	
H15A	0.0176	0.1391	0.2241	0.060*	0.50
H15B	−0.0176	0.1391	0.2759	0.060*	0.50

### Atomic displacement parameters (Å^2^)

**Table:**

	*U*^11^	*U*^22^	*U*^33^	*U*^12^	*U*^13^	*U*^23^
O1	0.109 (2)	0.092 (2)	0.0608 (15)	−0.0379 (16)	0.0301 (15)	−0.0327 (13)
O2	0.0605 (14)	0.0580 (14)	0.0726 (15)	−0.0154 (11)	0.0015 (12)	−0.0059 (12)
O3	0.0756 (15)	0.0679 (16)	0.0653 (15)	−0.0160 (12)	0.0091 (12)	−0.0159 (12)
O4	0.0658 (14)	0.0597 (14)	0.0748 (16)	−0.0169 (12)	0.0102 (12)	−0.0179 (13)
O5	0.0593 (14)	0.0617 (15)	0.0858 (16)	−0.0161 (12)	0.0222 (12)	−0.0191 (12)
N1	0.0513 (15)	0.0481 (15)	0.0543 (16)	−0.0003 (12)	0.0012 (13)	0.0053 (13)
C1	0.0496 (18)	0.0359 (16)	0.0527 (18)	−0.0006 (13)	−0.0027 (14)	−0.0012 (14)
C2	0.0597 (19)	0.0483 (18)	0.0543 (19)	−0.0076 (16)	0.0086 (15)	−0.0058 (16)
C3	0.059 (2)	0.0497 (19)	0.063 (2)	−0.0116 (16)	0.0148 (16)	−0.0048 (16)
C4	0.0456 (17)	0.0362 (17)	0.0578 (19)	0.0010 (13)	−0.0004 (15)	0.0018 (15)
C5	0.0593 (19)	0.0459 (18)	0.0552 (19)	−0.0024 (16)	0.0061 (15)	−0.0058 (15)
C6	0.0499 (18)	0.0527 (19)	0.063 (2)	−0.0059 (16)	0.0087 (15)	0.0009 (17)
C7	0.0475 (18)	0.0449 (18)	0.065 (2)	−0.0004 (14)	0.0028 (16)	0.0041 (17)
C8	0.0513 (18)	0.0350 (16)	0.061 (2)	0.0017 (14)	0.0033 (16)	−0.0043 (15)
C9	0.0450 (17)	0.055 (2)	0.061 (2)	−0.0051 (15)	0.0094 (15)	0.0089 (17)
C10	0.0518 (18)	0.0529 (19)	0.0509 (18)	0.0026 (15)	0.0093 (15)	0.0022 (15)
C11	0.0465 (17)	0.0364 (16)	0.0508 (17)	0.0044 (13)	0.0008 (14)	0.0045 (14)
C12	0.0504 (18)	0.064 (2)	0.059 (2)	−0.0045 (16)	0.0137 (15)	−0.0006 (17)
C13	0.062 (2)	0.058 (2)	0.0532 (19)	−0.0054 (17)	0.0070 (16)	−0.0081 (16)
C14	0.0467 (17)	0.0411 (17)	0.0607 (19)	−0.0015 (14)	0.0010 (14)	0.0004 (15)
C15	0.049 (2)	0.038 (2)	0.061 (3)	0.000	0.002 (2)	0.000

### Geometric parameters (Å, °)

**Table:**

O1—C2	1.358 (3)	C5—C6	1.391 (4)
O1—H1	0.8200	C5—H5	0.9300
O2—C7	1.271 (3)	C6—H6	0.9300
O2—H2	0.8200	C9—C10	1.383 (4)
O3—C7	1.246 (4)	C9—H9	0.9300
O4—C8	1.283 (3)	C10—C11	1.387 (4)
O4—H4	0.8200	C10—H10	0.9300
O5—C8	1.244 (3)	C11—C12	1.389 (4)
N1—C13	1.320 (4)	C11—C14	1.507 (4)
N1—C9	1.328 (4)	C12—C13	1.369 (4)
C1—C6	1.386 (4)	C12—H12	0.9300
C1—C2	1.396 (4)	C13—H13	0.9300
C1—C7	1.501 (4)	C14—C15	1.530 (3)
C2—C3	1.386 (4)	C14—H14A	0.9700
C3—C4	1.380 (4)	C14—H14B	0.9700
C3—H3	0.9300	C15—C14^i^	1.530 (3)
C4—C5	1.389 (4)	C15—H15A	0.9700
C4—C8	1.489 (4)	C15—H15B	0.9700
			
C2—O1—H1	109.5	N1—C9—C10	121.7 (3)
C7—O2—H2	109.5	N1—C9—H9	119.2
C8—O4—H4	109.5	C10—C9—H9	119.2
C13—N1—C9	119.3 (3)	C9—C10—C11	120.0 (3)
C6—C1—C2	118.9 (3)	C9—C10—H10	120.0
C6—C1—C7	121.2 (3)	C11—C10—H10	120.0
C2—C1—C7	119.9 (3)	C10—C11—C12	116.6 (3)
O1—C2—C3	119.6 (3)	C10—C11—C14	121.8 (3)
O1—C2—C1	120.4 (3)	C12—C11—C14	121.4 (3)
C3—C2—C1	119.9 (3)	C13—C12—C11	120.2 (3)
C4—C3—C2	120.6 (3)	C13—C12—H12	119.9
C4—C3—H3	119.7	C11—C12—H12	119.9
C2—C3—H3	119.7	N1—C13—C12	122.3 (3)
C3—C4—C5	120.4 (3)	N1—C13—H13	118.9
C3—C4—C8	118.6 (3)	C12—C13—H13	118.9
C5—C4—C8	121.0 (3)	C11—C14—C15	109.9 (2)
C4—C5—C6	118.8 (3)	C11—C14—H14A	109.7
C4—C5—H5	120.6	C15—C14—H14A	109.7
C6—C5—H5	120.6	C11—C14—H14B	109.7
C1—C6—C5	121.5 (3)	C15—C14—H14B	109.7
C1—C6—H6	119.3	H14A—C14—H14B	108.2
C5—C6—H6	119.3	C14—C15—C14^i^	113.7 (3)
O3—C7—O2	124.0 (3)	C14—C15—H15A	108.8
O3—C7—C1	120.0 (3)	C14^i^—C15—H15A	108.8
O2—C7—C1	116.0 (3)	C14—C15—H15B	108.8
O5—C8—O4	122.8 (3)	C14^i^—C15—H15B	108.8
O5—C8—C4	120.2 (3)	H15A—C15—H15B	107.7
O4—C8—C4	117.0 (3)		
			
C6—C1—C2—O1	178.6 (3)	C2—C1—C7—O2	179.2 (3)
C7—C1—C2—O1	−1.8 (4)	C3—C4—C8—O5	0.9 (4)
C6—C1—C2—C3	0.4 (4)	C5—C4—C8—O5	−179.0 (3)
C7—C1—C2—C3	180.0 (3)	C3—C4—C8—O4	−179.1 (3)
O1—C2—C3—C4	−179.1 (3)	C5—C4—C8—O4	1.0 (4)
C1—C2—C3—C4	−0.8 (5)	C13—N1—C9—C10	1.2 (4)
C2—C3—C4—C5	0.3 (5)	N1—C9—C10—C11	0.7 (4)
C2—C3—C4—C8	−179.5 (3)	C9—C10—C11—C12	−2.3 (4)
C3—C4—C5—C6	0.6 (4)	C9—C10—C11—C14	173.2 (3)
C8—C4—C5—C6	−179.5 (3)	C10—C11—C12—C13	2.2 (4)
C2—C1—C6—C5	0.6 (5)	C14—C11—C12—C13	−173.4 (3)
C7—C1—C6—C5	−179.0 (3)	C9—N1—C13—C12	−1.4 (5)
C4—C5—C6—C1	−1.1 (4)	C11—C12—C13—N1	−0.3 (5)
C6—C1—C7—O3	178.2 (3)	C10—C11—C14—C15	−90.2 (3)
C2—C1—C7—O3	−1.4 (4)	C12—C11—C14—C15	85.1 (3)
C6—C1—C7—O2	−1.3 (4)	C11—C14—C15—C14^i^	176.1 (3)

Symmetry codes: (i) −*x*, *y*, −*z*+1/2.

### Hydrogen-bond geometry (Å, °)

**Table:**

*D*—H···*A*	*D*—H	H···*A*	*D*···*A*	*D*—H···*A*
O4—H4···O5^ii^	0.82	1.82	2.631 (3)	172
O2—H2···N1	0.82	1.75	2.568 (3)	174
O1—H1···O3	0.82	1.79	2.516 (3)	147

Symmetry codes: (ii) −*x*+1, −*y*+4, −*z*+1.
